# *Glossolomawiehleri* (Gesneriaceae), a new species from the northwestern Andes of Ecuador

**DOI:** 10.3897/phytokeys.186.73554

**Published:** 2021-11-29

**Authors:** John L. Clark, Francisco Tobar

**Affiliations:** 1 Science Department, The Lawrenceville School, Lawrenceville, NJ 08648. U.S.A. The Lawrenceville School Lawrenceville United States of America; 2 Área de Investigación y Monitoreo de Avifauna, Aves y Conservación – BirdLife en Ecuador, Quito, Ecuador Aves y Conservación – BirdLife en Ecuador Quito Ecuador; 3 Instituto Nacional de Biodiversidad, Herbario Nacional del Ecuador QCNE, Quito, Ecuador. Instituto Nacional de Biodiversidad Quito Ecuador

**Keywords:** Ecuador, Gesneriaceae, *Glossoloma*, taxonomy

## Abstract

A new species of *Glossoloma* is described from the western Andean slopes of the Pichincha Province in northern Ecuador. *Glossolomawiehleri* J.L.Clark & Tobar is differentiated from all other congeners by an epiphytic habit, elongate scandent shoots that exceed four meters in length, and coriaceous leaves with a velutinous indument on the lower leaf surface. The new species is illustrated, featured with field images from recent expeditions, and assigned the category of Endangered (EN) according to IUCN Criteria.

## Abstract (fr)

Se describe una nueva especie de *Glossoloma* de las laderas occidentales de la provincia Pichincha en el norte de Ecuador. *Glossolomawiehleri* J.L.Clark & Tobar se diferencia de todos sus otros congéneres por su hábito epífito, ramas escandentes alargadas que superan los cuatro metros de longitud y hojas coriáceas con un indumento velutino en la superficie inferior de la hoja. La nueva especie se ilustra con imágenes de campo de expediciones recientes y se le asigna la categoría de En Peligro (EN) de acuerdo a los criterios de la IUCN.

## ﻿Introduction

The flowering plant family Gesneriaceae, with over 3400 species and 150+ genera (Weber 2004; Weber et al. 2013), is in the order Lamiales. The family is divided into three subfamilies and seven tribes (Weber et al. 2013, 2020), which represent monophyletic lineages (Ogutcen et al. 2021). The majority of New World members are in the subfamily Gesnerioideae and are represented by 1200+ species and 77 genera (Clark et al. 2020). *Glossoloma* Hanst. is classified in the tribe Gesnerieae and subtribe Columneinae (Weber et al. 2013, 2020).

The genus *Glossoloma* is defined by resupinate (upside down) flowers, a feature that was recently documented and discovered as a morphological synapomorphy (Clark and Zimmer 2003; Clark et al. 2006; Clark 2009; Clark et al. 2012). *Glossoloma* corollas are usually tubular, apically pouched and appear laterally compressed. Most *Glossoloma* are unbranched subshrubs with terminally clustered leaves, but some are scandent terrestrial subshrubs or facultative epiphytes with horizontal stems. The new species described here is unusual for its habit of being epiphytic with elongate horizontal scandent stems. Three other species in *Glossoloma* that have elongate scandent stems are *G.chrysanthum* (Planch. & Linden) J.L.Clark, *G.penduliflorum* (M.Freiberg) J.L.Clark, and *G.scandens* J.L.Clark. Several other species of *Glossoloma* are facultative epiphytes, but their stems are erect or non-scandent. Differences between *Glossolomawiehleri* and closely-related congeners are discussed below.

*Glossoloma* ranges from southern Mexico to Panama, northwestern South America, and south to Bolivia. The center of diversity of *Glossoloma* is the western lowland forests in Ecuador and Colombia where 15 species occur. *Glossoloma* was monographed by Clark (2009) and included 27 species. An additional species was described by Rodas and Clark (2014) from the Cordillera Central of the Colombian Andes. The description of *Glossolomawiehleri* brings the total number of *Glossoloma* species to 29.

## Taxonomic treatment

### Glossoloma wiehleri

Taxon classificationPlantaeLamialesGesneriaceae

J.L.Clark & Tobar sp. nov.

9D67398B-495E-5D1D-8ACF-25BCE423826F

urn:lsid:ipni.org:names:77233918-1

Figs 1 , 2

**Diagnosis**. Differs from all other congeners by the presence of elongate scandent shoots that exceed four meters in length, coriaceous leaves that are velutinous on lower surface, and a corolla tube that is broadly ampliate on the dorsal surface.

**Type. ECUADOR.** Pichincha: Quito towards Chiriboga, past San Juan and El Sigsal, kilometer #40, 27 Apr 1993, *H. Wiehler & Gesneriad Research Foundation Study Group 93228.* (holotype: SEL [095415]).

**Description**. Scandent subshrub with elongate horizontal shoots, sparingly branched, to 4 m long, to 1.5 cm in diameter, subwoody; internodes 3.8–8 cm long, subquandrangular, brown velutinous. Leaves opposite, isophyllous, coriaceous; blade symmetric, ovate to broadly ovate, 9.3–12.5 × 5–6.7 cm, base truncate to slightly cordate, apex acute, margin serrulate, adaxially light green, densely pilose, abaxially uniformly dark red to green with red venation, densely villous, lateral veins 6–9, primary and secondary veins occasionally red. Inflorescence reduced to a single axillary flower (rarely 2–3); peduncles absent or highly reduced (< 2 mm); bracts absent or caducous, 2 × 3 mm. Flowers resupinate, subtended by elongate pedicels, 1.2–4.5 cm long, densely pilose, oriented horizontal relative to shoot, becoming more pendent during anthesis; calyx lobes 5, nearly free, mostly equal in size and shape, dorsal lobe slightly smaller, lobes appressed to flower when immature and spreading during anthesis, mostly green with red margins, 1.6–3.0 × 1.0–1.3 cm, broadly ovate, apex acute to acuminate, margin with 5–10 deep serrations (ca. 4 mm long teeth), densely pilose on both surfaces; corolla tubular, broadly ampliate on dorsal surface (not ampliate on ventral surface), posture horizontal relative to calyx, corolla tube 2.4–2.6 cm long, outer surface densely pilose, uniformly bright yellow on the inside and white suffused with yellow on the outside, lobes 3–5 × 4–6 mm. Androecium of 4 stamens, filaments connate at the base and forming a filament curtain for 3–4 mm, free portion of filaments 2–2.5 cm long, glabrous; anthers longer than broad, ca. 2 × 1.5 mm, dehiscing by longitudinal slits; staminode lanceolate 3–5 × 1–2 mm; nectary a bilobed dorsal gland, sometimes appearing truncate, glabrous; ovary superior, densely pilose, 2–4 × 2 mm, style ca. 2.0 cm long, glabrous, stigma included and shallowly bifid. Immature fruit cone-shaped, densely pilose, 1.4 × 0.9 cm. Mature fruit not observed.

**Phenology.** Collected in flower during February, April, and July. Immature fruits observed in February.

**Etymology**. The specific epithet is in reference to Hans Wiehler (1930–2003). Wiehler was a practicing Mennonite from East Prussia (now Poland) and immigrated to the USA in the 1950s. He attained a Bachelor’s degree from the Eastern Mennonite College in 1954 and a Bachelor of Divinity degree in 1956 from Goshen College in Goshen, Indiana (Clark 2003). He eventually left the Mennonite church and pursued his passion for botany. Wiehler earned a Master’s degree from Cornell and obtained his Ph.D. in Botany from the University of Miami. Wiehler’s advanced degrees focused on the taxonomy and classification of Gesneriaceae. Wiehler was one of the first botanists hired by the Marie Selby Botanical Gardens where he served as the associate editor and business manager of the garden’s journal, *Selbyana* (1975–1981). He left Selby in 1982 and established the Gesneriad Research Foundation (GRF) in Sarasota, Florida where he directed annual seminars that were attended by horticulturists, taxonomists, students and plant enthusiasts. Wiehler also organized and directed 14 study trips to South and Central America, including the 1993 expedition that resulted in the discovery of *Glossolomawiehleri*. The first author met Hans Wiehler in 1994 and corresponded with him regularly until he died in 2003. Wiehler’s passion for Gesneriaceae was contagious.

**Distribution and preliminary assessment of conservation status.***Glossolomawiehleri* is endemic to the Pichincha Province on the western slopes of the Ecuadorian Andes and is known from three localities. The type locality is the old highway between Quito and Santo Domingo (via Chiriboga). In 2020, Tobar located an extant population of *G.wiehleri* near kilometer #40 (San Juan) where Hans Wiehler made the initial discovery in 1993 (Wiehler 1993). An additional population is supposedly from the Bombolí Cloud Forest, near kilometer #20 on the highway Quito–Santo Domingo (via Alóag). Clark facilitated a visit for Brian K. Schuetz in 2005 to the Smithsonian Institution’s National Herbarium (US). During that time, Schuetz was a graduate student at the Idaho State University (Pocatello, ID) and was completing research for his Master’s thesis on the woody flora of the Bombolí Cloud Forest. Schuetz had an unmounted specimen of *Glossolomawiehleri* (*B. Schuetz 600*) that was supposedly from 2955 m above sea level inside the Bombolí Cloud Forest. Schuetz’s dissertation (Schuetz 2004) provides longitude and latitude for most of his collections. *Glossolomawiehleri* is featured with images and a description (Schuetz 2004), but lacks detailed locality data (e.g. it is one of the only species in Schuetz’s floristic study that does not include longitude and latitude). Schuetz did not deposit specimens in an Ecuadorian herbarium. Likewise, specimens of *G.wiehleri* were not deposited at the Idaho Museum of Natural History (IDS). A third population was documented by Tobar in 2019 from the Bosque Protector Pacaya, a Reserve that is managed by the community Alaspungo. Bosque Protector Pacaya is adjacent to El Pahuma Orchid Reserve (Ceiba Foundation for Tropical Conservation). The forests in Bosque Protector Pacaya are mostly above 3,000 meters and the forests in El Pahuma Orchid Reserve are mostly below 3,000 meters. It is likely that populations of *G.wiehleri* are limited to forests above 3,000 meters and that is why it has not been documented in the lower elevation forests of El Pahuma Orchid Reserve. According to the IUCN Red List Criteria (IUCN 2012; IUCN Standards and Petitions Committee 2019) for limited geographic range (B1 = EOO < 5,000 km^2^) and associated subcriteria, including occurrence at less than five locations (B2a) and continuing decline of Andean forests (B2b), *Glossolomawiehleri* should be listed in the category Endangered (EN).

**Figure 1.**
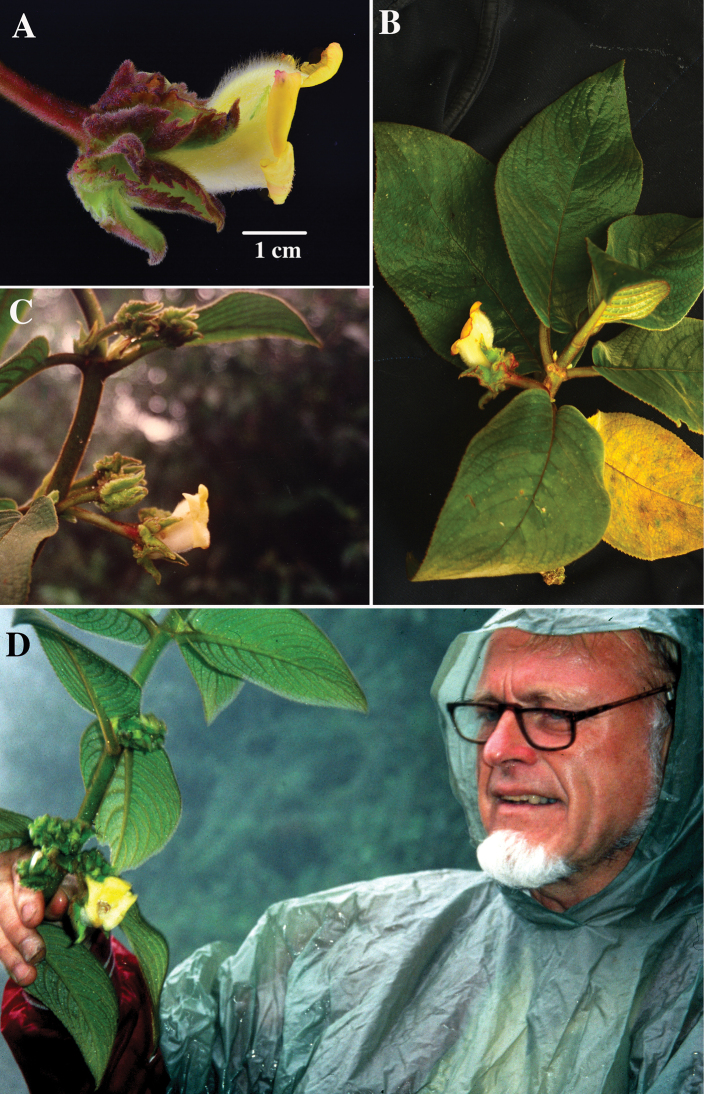
*Glossolomawiehleri* J.L. Clark & F. Tobar. **A** Mature flower **B** Stem with foliage **C** Stem with axillary clusters of flowers **D** Hans Wiehler holding the holotype (**A, B** from *Tobar & Gavilanes 3521***C, D** from *H. Wiehler et al. 93228*). Photos **A, B** by F. Tobar, **C** by M. Riley **D** by G. Robinson.

**Comments.** Most *Glossoloma* are terrestrial woody subshrubs with an unbranched primary stem. An epiphytic habit is unusual in *Glossoloma*, especially with elongate or scandent stems. *Glossolomachrysanthum, G.penduliflorum, G. scanden*, and *G.wiehleri* are the only known species of *Glossoloma* with an epiphytic habit and elongate scandent stems. Some species, such as *G.altescandens* (Mansf.) J.L.Clark or *G.herthae* (Mansf.) J.L.Clark are facultative epiphytes, but their stems are erect and non-scandent. The population of *Glossolomawiehleri* from the type locality was observed to have stems that exceed four meters in length. The longest recorded stem in the genus is *G.chrysanthum* that exceeded five meters in length (Fig. 3E). *Glossolomachrysanthum* is endemic to Venezuela and is differentiated from *G.wiehleri* by a corolla tube that is apically constricted (Fig. 3A) vs. apically ampliated (Fig. 1A). In addition, *G.wiehleri* differs by the presence of a velutinous indument on the lower leaf surface (vs. hirsute to pilose in *G.chrysanthum*) and coriaceous leaves (vs. papyraceous in *G.chrysanthum*). The mature resupinate flowers of *G.wiehleri* are inflated on the upper surface (i.e. ampliate or gibbous) and straight on the lower surface (i.e. not ampliate or gibbous). *Glossolomapenduliflorum* is readily differentiated from all other members of the genus by the presence of solitary axillary flowers with elongate slender pedicels that are 10–15 cm long, the longest known pedicels for any member of *Glossoloma. Glossolomascandens* differs from *G.penduliflorum* by the presence of three flowers per axil and relatively short pedicels (< 1 cm long).

**Figure 2.**
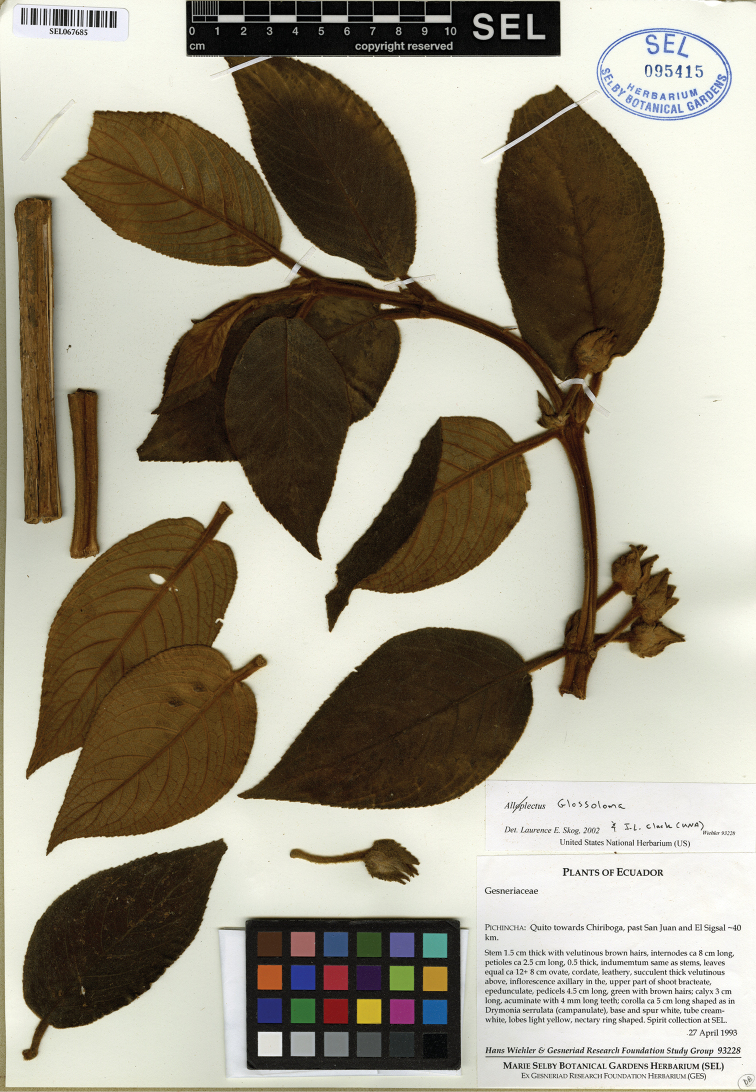
Holotype of *Glossolomawiehleri* J.L. Clark & F. Tobar (*H. Wiehler & Gesneriad Research Foundation Study Group 93228*, SEL).

**Specimens examined. ECUADOR. Pichincha**: cantón Quito, distrito Metropolitano de Quito, Chillogallo, road San Juan–Chiriboga, near San Juan, 0.416333°N, 78.6580°W, 3004 m alt., 20 Feb 2020, *Tobar & Gavilanes 3521* (QCNE); distrito Metropolitano de Quito, Nono, comunidad de Alaspungo, Bosque Protector Pacaya, 0.002320°N, 78.631260°W, 3000 m alt., 15 Jul 2019, *Tobar*, *Marcayata & Gavilanes 3399* (QCNE, US).

**Figure 3.**
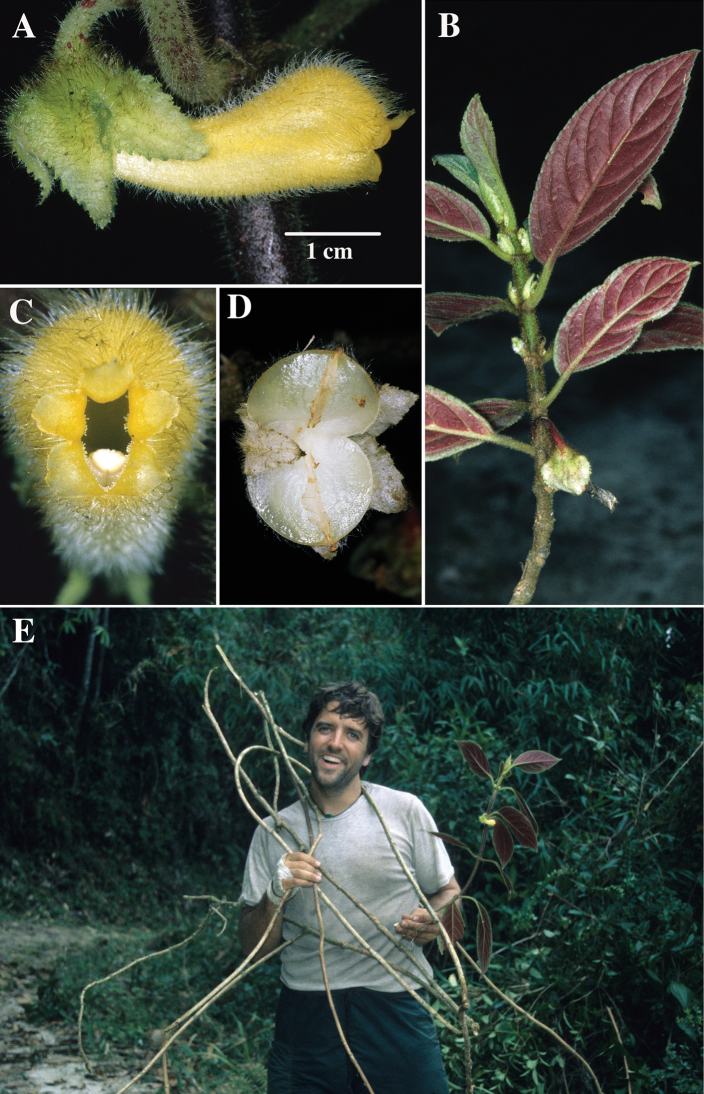
*Glossolomachrysanthum* (Planch. & Linden) J.L. Clark. **A** Mature flower **B** Stem with foliage **C** Front view of corolla **D** Mature capsule **E** Elongate scandent shoots held by John L. Clark during an exploratory research expedition in Venezuela (**A**–**E** from *J.L. Clark 6872*). Photos by J.L. Clark.

## Supplementary Material

XML Treatment for Glossoloma wiehleri
